# Polymer-Based Nanoparticle Strategies for Insulin Delivery

**DOI:** 10.3390/polym11091380

**Published:** 2019-08-22

**Authors:** Shazia Mansoor, Pierre P. D. Kondiah, Yahya E. Choonara, Viness Pillay

**Affiliations:** Wits Advanced Drug Delivery Platform Research Unit, Department of Pharmacy and Pharmacology, School of Therapeutic Sciences, Faculty of Health Sciences, University of the Witwatersrand, Johannesburg, 7 York Road, Parktown 2193, South Africa

**Keywords:** polymeric delivery systems, nanotechnology, insulin, bioavailability, biodegradable platforms

## Abstract

Diabetes mellitus (DM) is a chronic metabolic illness estimated to have affected 451 million individuals to date, with this number expected to significantly rise in the coming years. There are two main classes of this disease, namely type 1 diabetes (T1D) and type 2 diabetes (T2D). Insulin therapy is pivotal in the management of diabetes, with diabetic individuals taking multiple daily insulin injections. However, the mode of administration has numerous drawbacks, resulting in poor patient compliance. In order to optimize insulin therapy, novel drug delivery systems (DDSes) have been suggested, and alternative routes of administration have been investigated. A novel aspect in the field of drug delivery was brought about by the coalescence of polymeric science and nanotechnology. In addition to polymeric nanoparticles (PNPs), insulin DDSes can incorporate the use of nanoplatforms/carriers. A combination of these systems can bring about novel formulations and lead to significant improvements in the drug delivery system (DDS) with regard to therapeutic efficacy, bioavailability, increased half-life, improved transport through physical and chemical barriers, and controlled drug delivery. This review will discuss how recent developments in polymer chemistry and nanotechnology have been employed in a multitude of platforms as well as in administration routes for the safe and efficient delivery of insulin for the treatment of DM.

## 1. Introduction

Diabetes mellitus (DM) is a chronic metabolic illness estimated to have affected 451 million individuals to date, with this number expected to significantly rise in the coming years [1]. There are two main classes of this disease, namely type 1 diabetes (T1D) and type 2 diabetes (T2D) [2]. T1D is a result of autoimmunity, which is caused by the T and B cells of the immune system. These cells target and destroy the insulin-secreting β-cells situated in the pancreatic islets of Langerhans, resulting in a total lack of insulin secretion. T2D is largely due to a sedentary lifestyle, leading to resistance of the body’s tissues to insulin [3]. Diabetes is characterized by sustained hyperglycemia, which over time results in diabetic complications and eventually death. Blood glucose levels (BGLs) above 7.0 mmol/L during fasting and 11.1 mmol/L postprandial are indicative of diabetes.

Insulin therapy is pivotal in the management of diabetes, with diabetic individuals taking multiple daily insulin injections [2]. However, the mode of administration has numerous drawbacks, resulting in poor patient compliance. Insulin therapy aims to avoid fluctuations (hypoglycemia and hyperglycemia) in BGLs in order to prevent diabetic complications. However, it must be noted that insulin therapy cannot maintain BGLs at the same levels as physiological homeostasis in healthy individuals [3]. The ideal characteristics for insulin delivery include the real-time release of insulin in an appropriate dose response manner over a prolonged period of time, which current therapies do not satisfy. Therefore, there is a significant need for alternative insulin strategies.

In order to optimize different routes of insulin therapy, novel drug delivery systems (DDSes) have been suggested, and alternative routes of administration have been investigated. One such method used for the advancement of medicine is nanotechnology. Nanotechnology involves the use of particles within 1–100 nm. It is the size of these particles, as well as their large surface to volume ratio, that has increased interest in their application for molecular therapeutic targeting [4]. The use of nanoparticles (NPs) allows for improved bioavailability, controlled release, and targeted drug delivery (TDD). To date, the advancement of nanomedicine has focused on the safe, effective, and accurate delivery of drugs for an array of pathological conditions. Studies employing biodegradable natural/synthetic polymeric nanoparticles (PNPs) and manipulating the distinctive properties of these nanomaterials for TDD have been undertaken [5].

Advancements in nanoscience for insulin therapeutics have brought about research for the development of insulin nanocarriers, insulin smart-drug delivery systems (stimuli-responsive), insulin pumps, novel insulin analogs, and insulin nanosensors for the effective treatment of DM. Additionally, investigations into understanding the body’s architecture and physiological functions, including its communication, have taken place to determine how to achieve these systems biomimetically. This has brought about research into the development of a nanoresponsive polymeric artificial pancreatic system by incorporating polymer-based NPs with nanosystems. Hence, we can formulate insulin therapeutic platforms as desired to enhance the diabetic individual’s quality of life by using nanotechnological polymeric approaches [6].

This review will discuss how recent developments in polymer chemistry and nanotechnology have been employed in a multitude of platforms for the safe and effective delivery of insulin for the treatment of DM.

## 2. Polymeric Nanomaterials: Properties and Applications in Insulin Delivery

A novel aspect in the field of drug delivery was brought about by the coalescence of polymeric science with nanotechnology. The size of nanoparticle (NP) formulations for drug delivery should be considered within 100–1000 nm, since the nanoformulation contains a carrier and an active pharmaceutical ingredient [7]. The ability of polymeric nanomedicines to achieve TDD is influenced by its size, molecular weight, and surface charge. The size of PNPs affects their ability to cross physical barriers and arrive at the target site [8]. Additionally, PNPs need to remain in systemic circulation for TDD, while maintaining their surface charge [9]. Thus, PNPs can be modified to avoid rapid opsonization from the body [10]. To achieve a prolonged release of the drug in systemic circulation, a higher molecular weight of the polymer is required [11]. PNPs assist in improving bioavailability and biocompatibility, while maintaining the therapeutic efficacy of the drug.

In addition to PNPs, alternative routes of administration are also being investigated in order to optimize insulin delivery and move away from conventional insulin injections, thereby improving patient compliance. However, these noninvasive routes of administration are faced with challenges such as poor bioavailability, insufficient penetration of drugs through physical and chemical barriers, and the administration of open loop systems, resulting in poor regulation of BGLs [12]. By using polymer chemistry and nanotechnology in combination with different routes of administration, the therapeutic efficacy of insulin can be significantly improved (Table 1).

**Figure 1:**
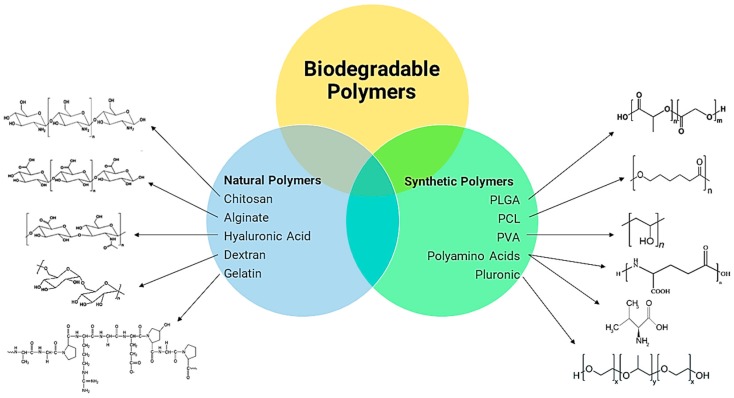
Biodegradable polymers employed in the delivery of nano-insulin formulations.

In this section, we seek to provide a review of natural and synthetic polymers, as outlined in Figure 1, that are used for nano-insulin delivery (their properties and routes of administration).

### 2.1. Natural Polymers

Natural or nonsynthetic polymers stem from nature and have brought about immense interest in the development of “green” research. Accordingly, these polymers are biocompatible, meaning they can be completely broken down by micro-organisms [12]. They also display low toxicity levels, thus attracting their application in insulin DDSes. Nonsynthetic polymers can consist of polysaccharides, including chitosan, alginate, hyaluronic acid, dextran; and proteins, such as gelatin. The use of chitosan in insulin DDSes has been well explored due to its abundance in nature and favorable characteristics, as discussed below.

#### 2.1.1. Chitosan (CS)

CS is derived from chitin, which is found on the shells of crustaceans, via alkaline deacetylation [42]. The structure of CS comprises *N*-acetyl glucosamine units that are positively charged, giving this polymer its mucoadhesive property [43]. CS also demonstrates biodegradability and nontoxicity and is inexpensive, thus making this polymer highly desirable for use in drug delivery research [44,45]. A study conjugating NPs and metals for insulin delivery significantly improved BGLs, as reported by Bhumkar et al. In this study, researchers developed CS/reduced gold NPs for the oral and nasal delivery of insulin. These administration routes are noninvasive and convenient; however, they present with challenges. Insulin administered orally passes through the liver to systemic circulation and becomes unstable and loses its therapeutic activity under gastric acidic pH. Insulin is also digested by proteolytic enzymes before reaching circulation [6,44]. The nasal mucosa serves as a barrier for insulin administration, as it does not allow large molecules to pass. Additionally, quick mucociliary clearance results in an insulin bioavailability of <1%. The formulated NPs by Bhumkar et al. were developed as insulin carriers and demonstrated a lowering of BGLs by 30.41% and 20.27% [13]. Additionally, a nanocomplex using chitosan as a nanocarrier was developed by Liu et al. [14]. Polyelectrolyte nanocomplexes were prepared based on CS-g-polyethylene glycol monomethyl ether (mPEG) copolymers through the self-assembly method for insulin delivery. In vitro and in vivo analysis was done to determine the mPEG graft ratio at which the best absorption could take place. The CS-g-mPEG copolymer was then compared to a fabricated mPEG-CS-glyceryl monocaprylate (GMC) copolymer to determine the influence of the hydrophilic surface modification for the absorption of oral insulin. The results demonstrated that enhanced absorption was attained, with an mPEG graft ratio of 10%, and that the mPEG-based copolymer presented with better permeation in the duodenum, along with having a better therapeutic effect when compared to the GMC-based copolymer. However, the results also demonstrated that the manipulation of CS-mPEG10% with GMC resulted in improved therapeutic efficacy. This study thus clarified the role of hydrophilic properties of insulin-loaded NPs and the effect of these on permeation through the intestinal mucus layer. Elsayed et al. developed insulin-loaded CS NPs for oral insulin administration. The CS NPs were formulated via the polyelectrolyte complexation (PEC) method, and the physicochemical characteristics of the formulated NPs were investigated. The results obtained demonstrated that the insulin-CS-PEC NPs were able to protect insulin from proteolytic destruction, thus delivering a therapeutic concentration of insulin over a 24-h duration [15]. At Nims University, India, researchers developed and assessed CS NPs for nasal administration of insulin. They developed a CS formulation that demonstrated a prolonged release of insulin from the NPs with a constant insulin plasma concentration, resulting in improved therapeutic efficacy [16]. Furthermore, Kondiah et al. investigated an immediate release, gastric absorptive oral insulin delivery system using a trimethyl chitosan (TMC) copolymer. Insulin-loaded polymeric particles showed a significant decrease in BGLs by 54.19% within 4 h (from the original blood glucose concentration). In vivo studies were evaluated using a diabetic New Zealand white (NZW) rabbit model [5]. These studies serve to confirm CS has an important role to play in insulin delivery due to its unique properties, allowing for insulin to be incorporated into novel DDSes as well as into alternative routes of administration, especially orally.

#### 2.1.2. Alginate

**Figure 2:**
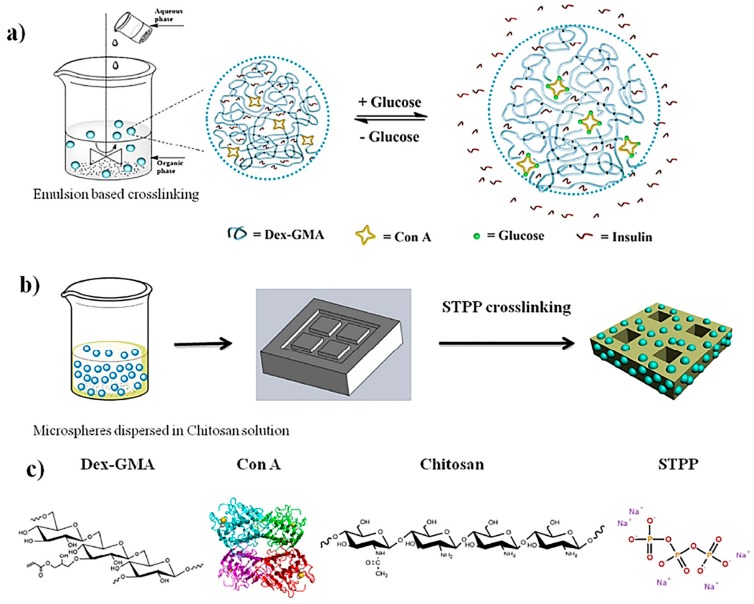
(**a**) Schematic process of fabricating ConA microspheres via ^w^/_o_ emulsion-based crosslinking, (**b**) preparation of the chitosan-based scaffolds for insulin delivery, and (**c**) chemical structures of dextran glycidyl methacrylate (Dex-GMA), concanavalin-A (ConA), chitosan, and sodium tripolyphosphate (STPP) (reproduced with permission from Reference [49]).

**Figure 3:**
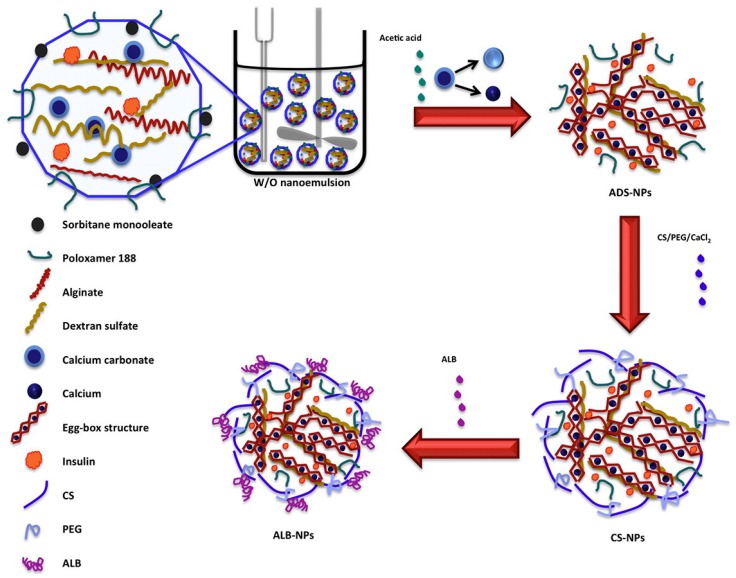
Schematic illustrating the fabrication of alginate-dextran NPs via a ^w^/_o_ nanoemulsion technique (reproduced with permission from Reference [23]).

Alginate, derived from seaweed, is composed of 1,4-linked β-d mannuronopyranosyl and α-l-guluronopyranosyl residues. Due to its carboxyl groups, alginate is polyanionic in nature, making it a polyelectrolyte responsive system [46]. Like CS, alginate possesses excellent biodegradability, nontoxicity, and mucoadhesion and low immunogenicity properties, resulting in the application of alginate for insulin drug delivery research [47]. If the environment has a low pH or divalent cations are present, alginate is able to form a gelling complex [46,48]. Studies carried out by Mansourpour et al. demonstrated the ability of alginate in combination with CS for insulin encapsulation. NPs were developed to incorporate the properties of both polymers, and β-cyclodextran was also added. This complexation allowed for the successful encapsulation of insulin and increased permeability for the oral absorption of insulin. However, further work will have to be carried out, as these studies were done in vitro [17]. A polyelectrolyte nanocomplex of alginate and CS was formed for the oral administration of insulin. This formulation had an association efficiency of 63% and a nanoparticle size of 748 nm. This study demonstrated that alginate–CS complexes show promise as insulin drug delivery carriers for oral therapeutic efficiency [18]. As alginate–CS nanocomplexes showed promise in vitro, researchers investigated coated alginate–CS–calcium chloride nanoemulsions (Figure 2a) containing insulin to determine their ability in oral delivery. An insulin entrapment efficiency was reported as 47.3%, and nanoemulsions ranged around 488 nm in size. The coated nanoemulsion was thus administered to diabetic rats, resulting in sustained blood glucose-lowering effects [19]. This technique can be observed in Figure 3 below, illustrating the fabrication of alginate-dextran NPs via a w/o nanoemulsion technique. 

**Figure 4:**
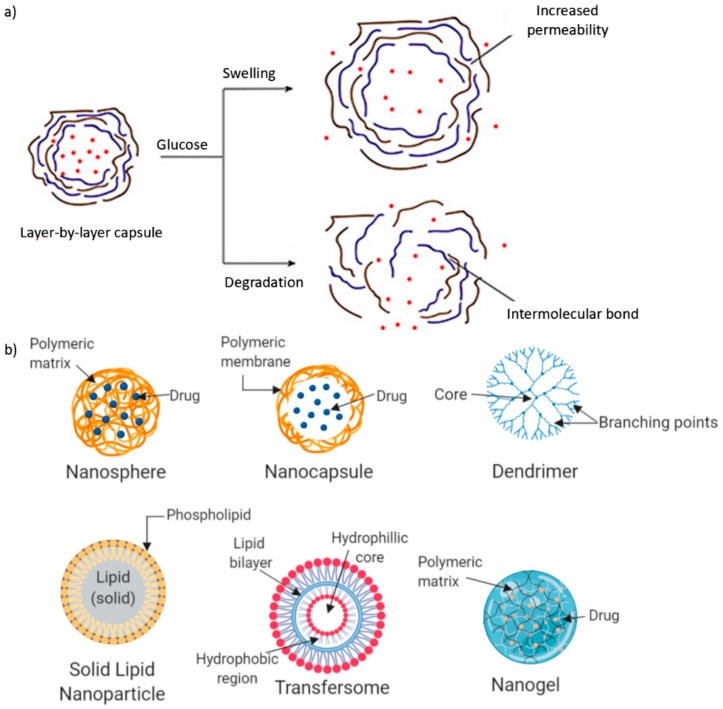
(**a**) Schematic illustrating the mechanism of layer-by-layer insulin nanoparticle capsules and (**b**) illustrating the nanosphere, nanocapsules, dendrimers, SLNs, transfersomes, and nanogel platforms for insulin loading and delivery (adapted with permission from Reference [81]).

Additionally, Verma et al. developed layer-by-layer calcium phosphate-coated NPs for the oral administration of insulin (Figure 4a). The researchers conjugated vitamin B12 (VB12) due to its pH responsiveness with CS and alginate polyelectrolyte polymers. In vivo studies demonstrated improved bioavailability as well as blood glucose-lowering effects over a 12-h duration [19,20]. The use of alginate in combination with CS both as nanocarriers and nanoencapsulation systems, forming PEC, demonstrated much promise. However, optimization studies need to be carried out in vivo to gain a better understanding of the effects and future potential of these alginate–CS PEC complexes in insulin delivery.

#### 2.1.3. Hyaluronic Acid (HA)

Hyaluronic acid, or hyaluronan, is a glycosaminoglycan made up of *N*-acetyl-d-glucosamine and glucuronic acid. HA is linked via unbranched alternating β-(1→3) and β-(1→4) glycosidic bonds [46]. Unlike CS and alginate, HA is negatively charged, and due to its stereochemistry, it is energetically stable. As a natural polymer, HA is both biocompatible and biodegradable, in addition to having low immunogenicity, and has thus garnered interest in its use in DDSes [50]. Liu et al. developed calcium carbonate-based nanocarriers that were coated with HA for the oral administration of insulin [21]. An effective blood glucose-lowering effect was obtained after oral administration to diabetic rats, in comparison to a subcutaneous injection of insulin. Studies carried out by Han et al. developed HA NPs for oral insulin administration. The HA NPs were pH-sensitive so as to protect the insulin from gastric and proteolytic degradation when administered orally. A size of 182.2 nm and 95% entrapment efficiency was achieved. The results illustrated an enhanced transport of insulin as well as hypoglycemic effects observed in vivo [22]. The oral administration of insulin is highly desired, and the use of HA to achieve this, as seen from the studies above, has significant potential. However, more studies need to be carried out both in vitro and in vivo before HA could be considered a competitor to CS and alginate polymers for insulin delivery.

#### 2.1.4. Dextran

Dextran is an exocellular bacterial polysaccharide that is water-soluble. It is a complex branched glucan, largely consisting of linear α-d-(1–6) glucopyranose linkages, with some degree of 1,3-branching. Dextran is biodegradable and biocompatible and has –OH groups that allow for a range of chemical manipulations to take place [44]. This hydrophilic and biocompatible polymer has been studied in combination with insulin for its pharmacokinetic and pharmacodynamic characteristics. Core-shell NPs were synthesized by Lopes et al. Insulin was enclosed in an alginate core coated with CS for the fabrication of insulin-loaded alginate/dextran sulfate NPs. The NPs were dual-coated with albumin and CS, as shown in Figure 3 below. Albumin acts as protection for the insulin molecule from proteolytic degradation and stabilizes the NPs in low and high pH environments. Data from the study demonstrated that 70% of insulin remained encapsulated within the NPs in simulated gastric fluid (SGF) with albumin as a coating. Additionally, a sustained release was obtained in simulated intestinal fluid (SIF) with surface albumin on NPs, enabling interaction with epithelial cells and enhancing insulin permeability [23].

Researchers in Iran prepared dextran– poly-lactic-co-glycolic acid (PLGA) NPs for the oral administration of insulin. This was carried out by blending an aqueous solution of insulin with the copolymer. Self-assembly took place, with various-sized polymersomes forming. The results showed that the NPs had an encapsulation efficiency and loading capacity of 90% and approximately 30%, respectively. This was at a 10:3 dextran–PLGA to insulin ratio. The in vitro permeability of dextran–PLGA NPs was greater than that of free insulin. The bioavailability of the NPs was reported as greater than free insulin, at 9.77% and 0.62%, respectively. This was at an administered dose of 100 IU/kg [24]. In India, Chalasani and coworkers aimed to fabricate VB12 and intrinsic factor ligand conjugates on dextran NPs for peroral insulin administration. In order to optimize the formulation, multiple molecular weights of dextran were used via emulsion. The insulin entrapment efficacy was 45%–70%, with insulin being protected from physical and chemical degradation by 65%–83%. Dextran at 70 K was seen to have the best results [51]. Further investigations were then carried out for design improvements to the VB12–dextran NPs in vivo. Streptozotocin (STZ) was used to induce diabetes in rats, with a 70%–75% BGL lowering being seen along with a pharmacological bioavailability of 29.4% and 54 h of antidiabetic effect in the animals [25]. These results suggest that the VB12–dextran complex could be an excellent platform for oral insulin administration.

#### 2.1.5. Gelatin

Gelatin is a protein polymer that is widely utilized in biomedical applications due to its biodegradable and nontoxic properties [48]. Gelatin has multiple functional groups, allowing for a plethora of chemical manipulations, and it has hydrophilic properties and is a polyampholyte. The physical and chemical modifications to gelatin depend on the crosslinking degree [52]. Glutaraldehyde crosslinked gelatin NPs were developed to provide a controlled release of insulin via swelling for oral administration. Since the acidic pH of the gastrointestinal (GI) tract causes insulin degradation, it was important that optimum release took place at an intestinal pH [26]. Moreover, researchers aimed to develop insulin-loaded NPs at a 1:1 ratio of gelatin/poloxamer 188. This was carried out to determine if these NPs could improve pulmonary insulin absorption and demonstrate improved pharmacological bioavailability. The inhalation of insulin has garnered interest due to pulmonary vascularization and the large surface area of the alveolar epithelium. This allows for the drug to quickly reach systemic circulation. However, pulmonary administration requires insulin to diffuse deep in the alveoli, possibly triggering an immune response, and if the surfactants are disturbed, proper respiration will not occur [53,54]. The fabricated gelatin–poloxamer NPs brought about reduced insulin deposition in the lung, which was needed to avoid an immune response; however, this was only observed a day after administration. The NPs demonstrated sustained lowering of BGLs as well as enhanced bioavailability [27]. Gelatin, a protein natural polymer, has demonstrated its ability for use in both oral and pulmonary insulin administration. Its ability to carry out controlled drug release, its protection against proteolytic and gastric pH degradation, and its enhanced absorption across mucosal membranes indicate its prospective use in the mucosal administration of insulin.

### 2.2. Synthetic Polymers

Synthetic polymers are hydrophobic and chemically and mechanically stronger in nature in comparison to their nonsynthetic counterparts. This mechanical strength reduces the degradation rate of the polymer, thereby providing the biomaterial with excellent durability [55]. By combining synthetic and nonsynthetic polymers, researchers are able to manipulate properties to achieve a superior insulin delivery system with enhanced therapeutic efficacy [4].

#### 2.2.1. PLGA (Poly-Lactic-co-Glycolic Acid)

PLGA is one of the most common drug encapsulation carriers and is used for its controlled release kinetics. Additionally, when hydrolysis takes place, PLGA breaks down and generates glycolic acid and lactic acid, which are metabolized naturally by the body. This results in a biodegradable system that is promising for insulin delivery [52]. Sateesh et al. developed 1.6% zinc insulin within PLGA, with iron oxide additives and fumaric anhydride oligomers, for oral insulin delivery. When compared to intraperitoneally administered zinc insulin, the NP formulation displayed an 11.4% greater efficacy, maintaining BGLs. The formulated PLGA insulin-loaded NPs demonstrated their ability to protect the molecular integrity of insulin during formulation and delivery. By using the solvent evaporation technique to synthesize NPs, 5% target loading and 75% higher encapsulation efficiency were observed. The yield of NPs fabricated by different methods varied from 55% to 99%. A burst release was seen in NPs synthesized by the solid o/w technique, and the sizes of NPs and the encapsulation efficiency were lowered by 223–243 nm and 0.3%–12%, respectively [28]. Researchers in China aimed to develop PLGA NPs for the administration of insulin orally. The formulated NPs demonstrated no burst release in an acidic pH, with insulin being released over 11 days. In vivo studies were also carried out and demonstrated a glucose-lowering effect that was able to be maintained [29]. Studies have also demonstrated that PLGA-conjugated CS NPs demonstrated desired effects in the oral administration of insulin [30]. By using CS to surround alginate, NPs were able to diffuse through the intestine due to their mucoadhesive properties and small size. The NPs also demonstrated a pH-dependent release, thereby enhancing their therapeutic efficacy [56]. Additionally, research carried out at the Huazhong University of Science and Technology showed that PLGA NPs loaded with insulin and contained within a polyvinyl alcohol (PVA) hydrogel were developed as a possible protein delivery platform. Data demonstrated an encapsulation efficiency of 72.6%, while in vivo studies carried out on STZ-induced diabetic mice showed blood glucose-lowering effects that were maintained over 24 h [31]. PLGA is a versatile synthetic polymer with the ability to form a complex/conjugate with both natural and synthetic polymers as well as metals. However, the burst release effect of PLGA insulin delivery systems in certain environments needs to be properly evaluated before it can be used to achieve an ideal insulin DDS.

#### 2.2.2. PCL (Poly-ε-Caprolactone)

PCL is a biodegradable polymer due to the hydrolysis of its ester linkages. Hydrolysis takes place under physiological conditions, thus garnering interest for use in DDSes. PCL is degraded over a period of time, increasing its potential for application in insulin delivery. The main synthesis methods for PCL NPs are solvent evaporation, nanoprecipitation, and solvent displacement [57]. Studies carried out at the State University of Campinas, Brazil, using insulin-loaded PCL NPs demonstrated that PCL NPs were biocompatible and had an insulin encapsulation efficiency of 90.6%. Animal studies demonstrated that the nanoparticle formulation maintained low BGLs, proving a controlled release system [32]. Researchers in France investigated the ability of PCL-blended polycationic acrylic NPs for oral insulin administration. The results demonstrated an encapsulation rate of 96%. These carriers, used for oral insulin delivery, provided a significant sustained hypoglycemic effect in the control and diabetic animal groups, which could have been a result of PCL’s mucoadhesive nature. Additionally, it was shown that the NPs were able to protect the insulin-loaded molecule [33]. Furthermore, Wu et al. developed an insulin-loaded nanoparticle formulation consisting of pH-sensitive polymers: poly (ethylene glycol)-PCL-poly (*N*, *N*-diethylamino-2-ethylmathaerylate). The mPEG-PCL-PDEAEMA was formed by atom transfer radical polymerization and ring opening polymerization, while the NPs containing insulin were formulated by nanoprecipitation. The nanoparticle formulation had an entrapment efficiency of 81.99% and exhibited low release kinetics, which were maintained with BGLs over 48 h [34]. Although PCL is able to form an NP for insulin drug delivery, this system may be improved by the incorporation of other polymers and/or metals. The slow degradation of PCL will allow for a controlled release of insulin over a time period, and data have demonstrated that PCL nanosystems have a high encapsulation and entrapment efficiency on insulin: by using PCL in a stimuli-responsive system, researchers may be able to attain an ideal insulin DDS.

#### 2.2.3. Polyvinyl Alcohol (PVA)

PVA is a biodegradable and biocompatible polymer with low toxicity and thermal stability. Like most synthetic polymers, PVA has a high level of mechanical strength and is easy to prepare. PVA has the ability to blend with natural polymers, resulting in novel DDSes with enhanced properties [58]. CS–PVA polymers were crosslinked by Zu et al. using glutaraldehyde for the fabrication of nano-insulin-loaded hydrogels for transdermal administration. In vitro results demonstrated robust thermal and physical characteristics with a high permeation, suggesting that the nano-insulin-loaded hydrogels are a promising noninvasive TDD system [35]. Rawat et al. prepared PVA NPs via solid ^o^/_w_ emulsion while optimizing the surfactant in am aqueous phase medium. The results showed that high-molecular-weight PVA demonstrated good chemical and physical properties for the stabilization and protection of insulin. Animal studies further evidenced this by retaining insulin bioactivity as well as by showing hypoglycemic effects [36]. PVA can also be used as a stabilizing surfactant in the fabrication of NPs for oral insulin administration [59].

#### 2.2.4. Polyamino Acids

Amino acids are some of the most imperative building blocks in biological systems. Their unique characteristics determine their structure and function. As opposed to natural polymers, synthetic-containing amino acid side-chain polymers are easy to fabricate, offering properties needed in various biomedical applications [60]. CS/poly-g-glutamic acid (CS/gPGA) NPs were fabricated by heating PGA acid with CS in solution for peroral insulin administration. This reaction was carried out in the presence of MgSO_4_ and tripolyphosphate (TPP). The results demonstrated that the NPs had a blood glucose-lowering effect for 10 h in vivo, while insulin bioavailability was 15.1%. In contrast to CS/gPGA NPs, TMC/gPGA NPs may be a suitable carrier for the transmucosal delivery of insulin within the entire intestinal tract, where the pH values are close to the pKa of CS [37]. Sonaje et al. developed a gelatin enteric-coated CS-poly(γ-glutamic acid) nanoparticle system for the oral administration of insulin. The gelatin coating served to protect the insulin-loaded NPs from acidic and enzymatic degradation. It was thus reported that insulin was efficiently delivered to the small intestine, with a sustained hypoglycemic effect from loaded insulin observed [38]. Scientists in China developed CS-based carboxyl-, PBA-modified and L-valine (LV) (CMCS-PBA-LV) multifunctional NPs for the oral administration of insulin. In vitro results showed little to no toxicity and efficient glucose-responsive properties, while data from animal studies demonstrated significant oral delivery and an effective hypoglycemic result in vivo [39].

#### 2.2.5. Pluronics

Pluronics (or poloxamers, as they are commonly referred to) are triblock polymers that are amphiphilic and water-insoluble in nature [61]. They are made up of polypropylene oxide with polyethylene oxide blocks on either side (PEO-PPO-PEO). Pluronics are tasteless, odorless, and waxy white granules that have thermosensitive gelling properties and are biodegradable. They are categorized according to their physical state (i.e., solid, paste, or liquid form) and their molecular weights [62]. Shu et al. carried out in vitro and in vivo studies to determine the effect of poly(lactic acid)-b-pluronic-b-poly (lactic acid) NPs carrying insulin for oral administration. The results displayed effective oral insulin absorption from the nanoparticulate delivery system [40]. Meanwhile, Xie et al. investigated the therapeutic efficacy of folic acid-pluronic 85-poly(lactide-co-glycolide) on orally administration insulin. The team developed pluronic F127-grafted PLA NPs for the oral administration of insulin, with in vitro studies exhibiting the biphasic release of insulin. In vivo BGLs were demonstrated within 4.5–5 h after administration, with BGLs being maintained for over 18.5 h [41]. Due to their robust thermal sensitivity, pluronics can be used in the development of injectable gels. Under physiological conditions, pluronics will gel, helping to form a drug-loaded deposit in the body. The pluronic matrix will also allow for the controlled release of insulin. By incorporating pluronics as a nanoparticulate platform (such as a nanogel) or for nanoencapsulation or as a nanocarrier for insulin delivery, researchers may incorporate a system that allows for the sustained stimuli-responsive delivery of insulin.

Although showing much promise, in addition to PNPs, a multitude of systems have been developed at a nanomeric scale for the purpose of achieving an ideal insulin DDS. These nanosystems will be discussed below.

## 3. Nanoplatforms and Their Properties for Nano-Insulin Delivery

In addition to PNPs, insulin DDSes can incorporate the use of nanoplatforms/carriers. A combination of these systems can bring about novel formulations and lead to significant improvements in the DDS with regard to insulin therapeutic efficacy, bioavailability, increased half-life in body circulation, better transport through physical and chemical barriers, and the development of the desired release profiles. Nanoplatforms have been developed for the delivery of insulin through various forms of tablets, gels, films, and needles. These serve as alternatives to the conventional insulin injections, which have unwanted side effects. The choice of nanoplatform depends significantly on the desired administration route and release profile [30]. Table 2 discusses the insulin routes of administration, with advantages and disadvantages for each, as well as examples of the nanoplatforms used to deliver insulin.

These nanoplatforms can be classified by using the size of their formulation, form of drug carrier, and core material. NPs are smaller than 100 nm. Nanocarriers can take the form of NPs, which are made up of nanospheres and nanocapsules, dendrimers, solid lipid nanoparticles, transfersomes, and nanogels for insulin delivery, as seen in Figure 4b. The core materials include polymer-based NPs (Figure 1) and lipid-based NPs, of which only solid lipid nanoparticles are suitable for insulin delivery. Drug molecules can be dispersed, contained, conjugated, and absorbed within the NPs. TDD via NPs is possible due to their nanosize [80].

### 3.1. Nanoparticles (NPs)

**Figure 5:**
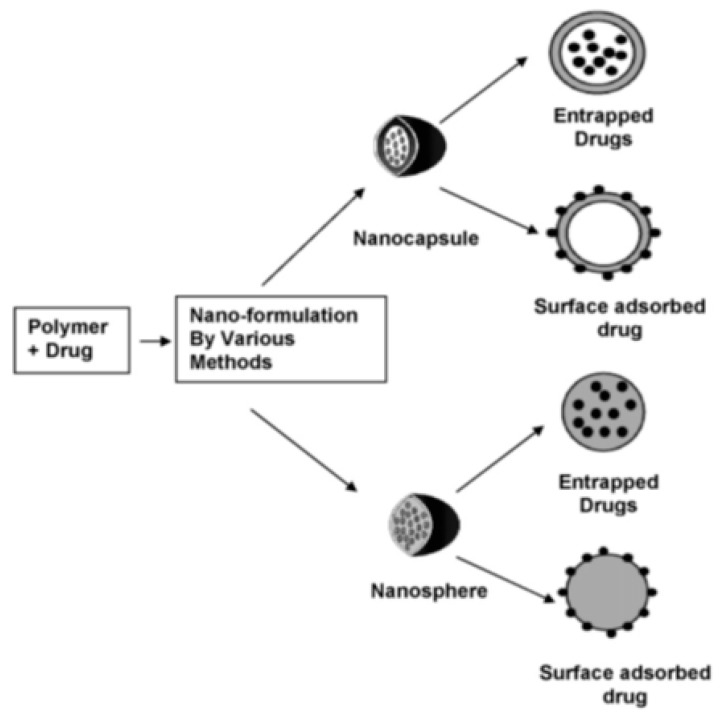
Differential morphology of the types of NPs employed for polymeric insulin delivery (reproduced with permission from Reference [52]).

NPs are solid particles that can be made up of polysaccharides, lipids, metals, and polymers [82]. Based on form and synthesis, NPs can be divided into nanocapsules and nanospheres, as shown in Figure 5. Nanocapsules can contain drugs within their empty shell, while in nanospheres, drugs are dispersed in a matrix of the particle [83]. Nanocapsules and nanospheres are nanocarriers that are generally referred to as NPs. Moreover, these NPs are able to conjugate to compounds such as cell-penetrating peptides (CPPs) to facilitate the transduction of proteins such as insulin into cells by permeating the plasma membrane [84]. CPPs are short peptide sequences (<30 amino acids) that can enhance the rate of absorption of therapeutic proteins and can be formulated in DDSes to improve the pharmacokinetics (absorption, distribution, metabolism, and excretion) of therapeutic proteins [85]. In addition to this, mesoporous silica NPs (MSNs) have been investigated for their robust physical and chemical characteristics. Due to their porosity, they also possess a large drug-loading capacity and are easily manipulated [86].

In addition to polymers in nanosystems, natural supplements have also shown promising antidiabetic results. HCD (16-hydroxycleroda-3, 13-dine-16, 15-olide) comes from the *Polyalthia longifolia* plant, native to India. Studies carried out by Huang et al. showed that this HCD extract could be used for surface modifications to MSNs, and the results showed that the MSN–HCD NPs were effective in treating diabetes in vivo [87]. Researchers in Shanghai, China, used biomimetic principles to design NPs that exploited the intestinal bile acid pathway to enhance the absorption of therapeutic proteins. They synthesized deoxycholic acid-conjugated CS loaded with insulin into deoxycholic acid-modified NPs. In vivo studies in diabetic rats showed notable hypoglycemic effects with an enhanced bioavailability of the NPs [88]. Meanwhile, Deng et al. formulated selenium (Se) NPs loaded with insulin. This was synthesized by ionic crosslinking/in situ reduction to overcome the intestinal absorption barrier. Their results demonstrated that the formulated SeNPs could effectively encapsulate insulin and improve its stability in gastric acid, showing a high hypoglycemic effect in diabetic rats following oral administration. The antidiabetic effect of Se as an insulin carrier contributed to the hypoglycemic effect of the insulin SeNPs [63]. Using insulin as a model protein, Niu et al. designed nanocapsules composed of an oily core consisting of oleic acid, sodium deoxycholate (SDC), and a polyarginine (PARG) shell. Their results showed that the prepared nanocapsules increased insulin absorption across intestinal epithelial cells, and in vivo experiments indicated that PARG nanocapsules had the ability to facilitate the interaction of protein and penetration enhancers with intestinal epithelial cells. Starch NPs were also investigated and prepared by Jain et al. for the nasal administration of insulin. This mucosal route allows for relatively easy access of insulin to systemic circulation due to the rapid blood flow and large absorption surface area of the nasal mucosa [64]. The insulin-loaded starch NP formulation that contained permeation enhancers demonstrated sustained hypoglycemia for up to 6 h. However, further studies need to be carried out to develop a viable nasal carrier [74]. In addition, recently, next-generation microneedle systems have been developed for the transdermal application of insulin. The transdermal delivery of insulin avoids both liver metabolism and the GI tract and is easy to administer. In this study, microneedles were designed to carry and release insulin from stimuli-responsive NPs. Hypoxia-responsive polymers were used to develop the NPs and were reduced in hypoxic microenvironments. Once reduced, the particles subcutaneously degraded, and release insulin was observed [53]. Thwala et al. formulated two polymer nanocapsules by using protamine for oral insulin delivery. Protamine is a cationic polypeptide that can be used as an oral drug delivery carrier for insulin due to its cell-penetrating properties [64,89]. The first nanocapsule shell consisted of a single layer of protamine. The second shell consisted of a double protamine/polysialic acid layer. The nanocapsules were evaluated in a simulated intestinal media containing proteolytic enzymes and bile salts. Data from this study demonstrated a controlled release of insulin, the preservation of insulin drug bioactivity, and significant glucose-lowering effects from the nanoplatform.

With regard to CPPs, Niu et al. developed an oral peptide delivery nanocomplex between insulin and octa-arginine R8 (the selected CPP) enveloped by polyglutamic acid–polyethylene glycol (PGA–PEG) in order to avoid enzymatic degradation. The CPP was chemically altered to promote insulin interaction. The results demonstrated that the formulated system was stable in intestinal fluid and that insulin was adequately protected from degradation. Their system also demonstrated adequate diffusion through intestinal mucus, as well as the successful transport of insulin orally [65]. Additionally, a CPP, SAR6EW, was investigated by Li et al., and the formulated NPs were compared to NPs without CPP [66]. Using an ionic/ionotropic gelation technique, they prepared CS NPs carrying CPP and insulin. The improved bioavailability of insulin by the NPs and the toxicity of the NPs were evaluated. Their results presented that the formulated CPP complex had better enzymatic stability in comparison to the NPs without the CPP, along with having an improved cellular uptake profile. Following oral administration of the NPs to diabetic rats, a successful hypoglycemic effect and low cytotoxicity were reported. Another CPP, penetratin, was chemically conjugated to CS (CPP-g-Cs) by Barbari et al. to evaluate the synthesized NPs for the delivery of proteins and peptides orally. Insulin was loaded into the NPs using ionic gelation techniques, with the application of STPP as a crosslinking agent. These NPs were then put into enteric protective capsules, which were evaluated in vivo in rats to determine the effect on BGLs. The results showed that the synthesized NPs demonstrated a hypoglycemic effect in vivo for 10 h and should be further studied to enhance their capabilities as an oral drug delivery platform for proteins [67]. Scientists at Fudan University, China, developed CPP PLGA NPs for orally administered macromolecules. The NPs were functionalized with a levorotation and dextrorotation (L/D) configurations, as polyarginine-conjugated PLGA NPs. The enantiomers demonstrated a significant improvement in the intestinal absorption of insulin as well as hypoglycemic effects in comparison to unmodified PLGA NPs [68]. As such, NPs have shown an ability to be utilized in a variety of forms as well as in administration routes both in vivo and in vitro.

### 3.2. Dendrimers

Dendrimers are 3-D polymeric nanocarriers that consist of a core that can carry water-insoluble drugs and surface-active groups that help determine the properties of the dendrimer [5,43]. Polyamidoamine (PAMAM) is often used as a dendrimer due to its absorption-enhancing effects, which is important for the mucosal delivery of insulin. This characteristic may be due to PAMAM’s positive charge [90]. Researchers at the Department of Biopharmaceutics, Kyoto Pharmaceutical University, Japan, demonstrated that using 1% (w/v) G3 PAMAM dendrimers could improve insulin absorption via the nasal route of administration without damaging surrounding epithelial tissue [75]. These researchers, in collaboration with Hamid, Gao, and Lin, then investigated the pulmonary absorption effect of PAMAM dendrimers using insulin in vivo. The data demonstrated that absorption was concentration- and generation-dependent and that PAMAM dendrimers enhanced pulmonary insulin absorption significantly [76]. Thus, the use of dendrimers for alternative routes of administration shows much promise; however, significant work has to be carried out before clinical trial evaluation.

### 3.3. Solid Lipid Nanoparticles (SLNs)

SLNs are composed of lipid molecules such as waxes; mixtures of mono-, di-, and triacylglycerols; and fatty acids. They have a high biocompatibility and are feasible in production for large-scale synthesis [69]. In a study conducted by Boushra et al., the shortfalls of insulin delivery were sought to be overcome by developing a bipolymer lipid hybrid nanocarrier (BLN) system. In this study, PEG and PLGA polymers were used for incorporation into a double emulsion-based SLN for oral insulin delivery. The results demonstrated that the developed BLNs achieved a greater entrapment of insulin, while having low cytotoxicity, and were able to protect the entrapped insulin under gastric conditions. In vivo studies demonstrated blood glucose-lowering effects with oral administration of BLNs [69]. At the University of Porto, Portugal, Sarmento et al. investigated the possibility of cetylpalmitate SLNs in orally delivering insulin. The cetylpalmitate SLNs were evaluated in vivo and presented significant blood glucose-lowering in comparison to subcutaneous insulin over 24 h. These results suggest that SLNs protect insulin from enzymatic degradation and acidic pH, consequently enhancing intestinal absorption of insulin in vivo [70]. Furthermore, SLN flocculate insulin carriers were developed by Yang et al. for inhaled insulin administration. The positively and negatively charged Ins-SLNs were synthesized via water in oil water (w/o/w) emulsion, self-assembled via electrostatic interactions, and finally lyophilized. In vivo investigations also demonstrated a favorable hypoglycemic response with significant bioavailability of insulin [91]. Ansari et al. aimed to develop SLNs using glyceryltrimyristate, PVA, and soy lecithin. Following optimization and characterization, the results exhibited that the formulation protected insulin from the GI tract and had a 5× greater bioavailability than insulin solution did [92]. Finally, a collaboration between the Instituto Superior de Ciências da Saúde and the University of Porto led to investigations into the properties of insulin-loaded CS-coated Witepsol 85E SLNs for the oral administration of insulin. Animal studies demonstrated that the oral administration of the CS-coated SLNs had significant hypoglycemic effects and bioavailability in comparison to uncoated SLNs over 24 h [71]. SLNs have shown a remarkable ability to bypass barriers such as proteolytic enzymes and gastric pH degradation to achieve oral insulin administration. Along with their biocompatibility and cost feasibility, SLNs demonstrate promising platforms for oral insulin delivery.

### 3.4. Transfersomes

Transfersomes consist of water, phospholipids, and liposomes and are used to carry water-soluble drugs such as insulin. Transfersomes have a deformable morphology, demonstrate membrane flexibility, and assist in TDD via oral or transdermal routes of administration [93]. The transdermal administration of insulin can occur via diffusion through the intact skin barrier or by physically disrupting the skin barrier. As such, the skin is the main barrier to transdermal administration, with the stratum corneum being the first and most difficult to bypass [55]. Research carried out by Yang et al. used transfersome vehicles to determine their effect on insulin delivery in vivo via the buccal route. Buccal administration of drugs occurs on the inner cheek and has demonstrated a high absorption permeability of mucosal membranes; however, it is not without challenges. Insulin must pass through both the mucosal and epithelial layers to get to systemic circulation [53]. While in the presence of sodium deoxycholate (NaDC), transfersomes displayed an enhancing effect on insulin delivery when compared to subcutaneous insulin administration. The deformable transfersome vehicles carrying insulin also demonstrated a higher relative pharmacological bioavailability of 15.59% and a relative bioavailability of 19.78% [73]. The use of transfersomes has also displayed the ability to overcome the stratum corneum for transdermal insulin delivery, employing chemical enhancers. Studies by Cevc et al. indicated that BGLs were able to be lowered and maintained below 10 mm/L when insulin was administered transdermally [77]. Sharma et al. aimed to develop a platform for oral administration. Using electrospun PVA and sodium alginate nanofibers, investigators developed an insulin-loaded transmucosal patch. In vitro studies demonstrated a controlled drug release and an encapsulation efficiency of 99%, while data from animal studies showed that insulin was still active when delivered [94]. Researchers in India have assessed the ability of transfersome gels in the transdermal delivery of insulin. The results demonstrated that insulin had a 78% entrapment efficiency when a chemical enhancer was added to the gel, demonstrating hypoglycemic effects in comparison to the control gel [78]. Another study investigating the transdermal delivery of insulin using transfersomes was conducted by Malakar et al. in alloxan-induced diabetic rats. After insulin-loaded transfersome administration, it was observed that a hypoglycemic effect was maintained for 24 h [79]. Transfersomes have demonstrated an ability to administer insulin via multiple administration routes.

### 3.5. Nanogels

**Figure 6:**
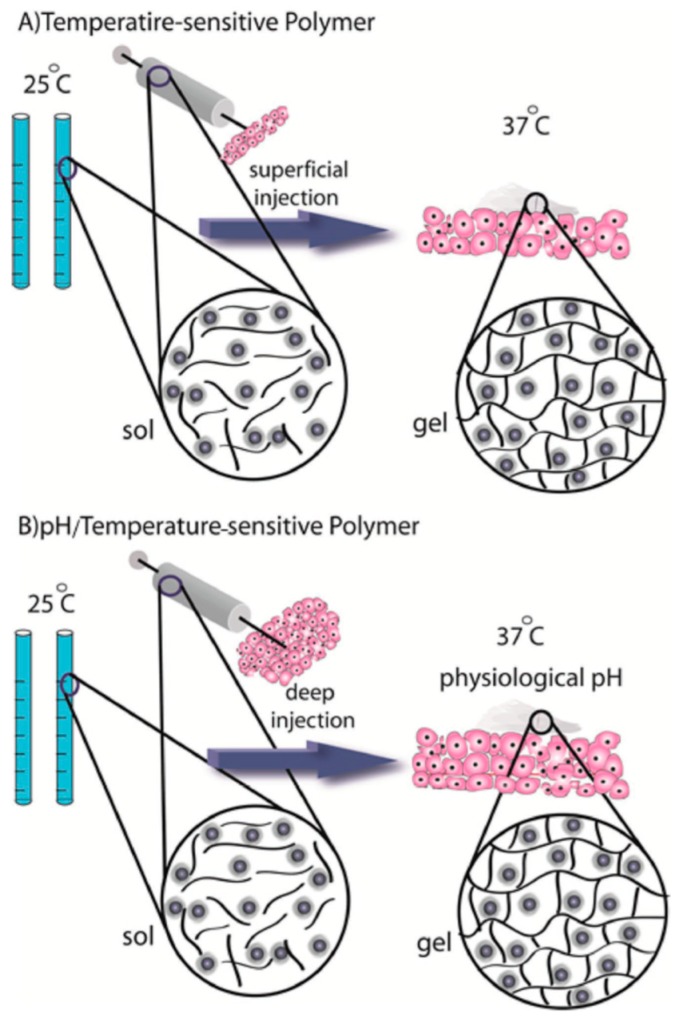
Illustration of stimuli-responsive mechanisms employing (**A**) temperature-sensitive polymer reactivity and (**B**) pH/temperature-responsive polymer reactivity undergoing sol–gel transition (adapted with permission from Reference [4]).

Nanogels are 3-D hydrogel materials on a nanoscale formed by crosslinked swellable polymer networks that are able to retain large amounts of water without being solubilized [95]. Nanogels have attracted increasing interest in DDSes due to their porosity, biocompatibility, charge, and ability to have chemical compositions altered [95,96]. The size of nanogels allows for intracellular TDD [97]. Nanogel fabrication can occur from the crosslinking of both natural and synthetic polymers. It is this polymer network that allows for the encapsulation of drugs, such as insulin, in the nanogel, while environmental factors may cause a stimuli-responsive sustained release of the drug [96]. Zhao et al. encapsulated insulin into nanogels sensitive to pH and temperature (Figure 6). The group synthesized hydroxypropyl methylcellulose (HPMC) nanogels without the need for a surfactant and thus with no organic solvent, making this a green environmentally friendly method. The drug loading was reported as 21.3%, with an entrapment efficiency of 95.7%. The system showed sustained insulin delivery, with insulin release from the nanogel responsible being pH- and temperature-dependent [98].

Wang et al. synthesized hollow nanogels via a two-step colloidal template polymerization. Template polymerization allows for the fabrication of a hollow structure that is favored due to its low density and larger area for drug loading [99]. Phenylboronic Acid (PBA) moieties were then introduced to the system. Insulin was encapsulated within the nanogel, thus protecting it from external degradation. The results demonstrated that the nanogels were successfully synthesized to poly(*N*–isopropylacrylamide) and poly(*N*-phenylboronic acid acrylamide) to give a Poly (NIPAM-AAm-PBA)] complex. The nanogel system demonstrated dual-responsiveness with regard to temperature and glucose stimuli [100]. In a more complex molecular design, researchers at Jilin Jianzhu University and the Changchun Institute of Applied Chemistry fabricated a glucose-responsive nanogel by synthesizing a methoxy poly(ethylene glycol)-block-poly(γ-benzyl-l-glutamate-co-(γ-propargyl-l-glutamate-graft-glucose)) or (mPEG-b-P (BLG-co-[PLG-g-Glu]) and a small molecule with a PBA group at both ends. Adipoylamidophenylboronic acid acted as a crosslinking agent to bind to the glucose end group of mPEG-b-P (BLG-co-[PLG-g-Glu]) to form a nanogel. The introduction of external glucose directly reduced the degree of crosslinking of the gel and caused the gel to swell, thereby releasing insulin to the stimuli [101].

Additional research in this area was carried out via emulsion polymerization to fabricate a hydroxyethyl methacrylate nanogel system. Insulin was encapsulated within the nanogel for oral insulin delivery. Using fluorescein isothiocyanate (FITC)–dextran, investigators were able to determine the enhanced intestinal absorption of insulin. In vivo studies showed a negligible immune response as well as an improved half-life and controlled BGLs for 24 h [72]. The ability to retain large amounts of water, mimicking human tissue, along with excellent biocompatibility, makes nanogels an ideal drug carrier for the delivery of insulin. These results indicate that nanogels could be an ideal insulin DDS carrier.

## 4. Controlled Release and Stimuli-Responsive Systems for Nano-Insulin Delivery

The main therapy for the treatment of diabetes is injectable insulin administered subcutaneously; however, this has various shortfalls. Ideal insulin therapy should be easy to administer in an appropriate dose-responsive manner (stimuli-responsive) over a prolonged period of time. Thus, these systems bring together the emergence of smart responsive closed-loop delivery of insulin. There are two methods to achieve a closed-looped system: the traditional “artificial pancreas” or the “synthetic artificial pancreas”. The traditional pancreas is an external electronic device containing a continuous glucose monitor (CGM), while the synthetic or chemical pancreas is based on physiological physical and chemical stimuli and is administered as a drug formulation directly to the patient [49,102]. The synthetic pancreas is favored over the traditional artificial pancreas due to its improved therapeutic efficacy, small size, and minimally invasive implantable ability [49]. Over the last decade, a variety of natural biopolysaccharides have been exploited in the design and synthesis of many responsive DDSes. These systems may be activated to respond to changes such as pH, temperature, ionic strength, electric fields, magnetic fields, and ultrasound [103]. In this section, we will be focusing and discussing chemically responsive nanosystems that are being developed for the administration of insulin, as well as insulin release mechanisms [46,104].

**Figure 7:**
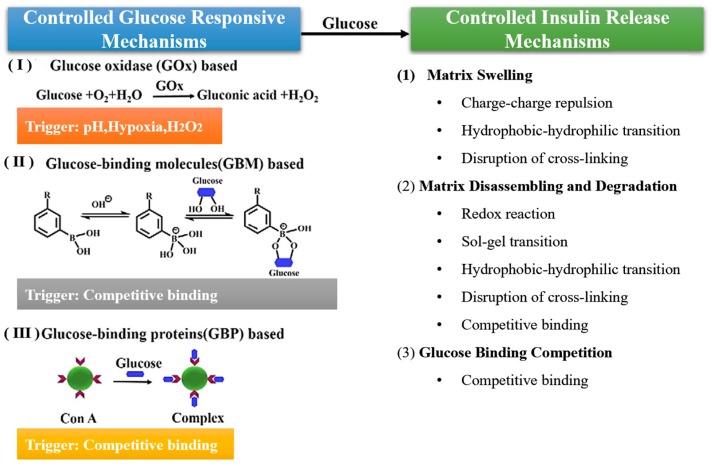
Illustration of the mechanisms employed to achieve chemically controlled closed-loop systems for insulin release (adapted with modifications from Reference [102]).

The main elements of a closed-loop system are a glucose-sensing element and a sensor-triggered insulin release element. Additionally, the glucose responsive insulin release (GRIR) system should spontaneously switch on and off as BGLs rise above 7.0 mmol/L (fasting) or 11.1 mmol/L (postprandial) and drop to normoglycemia at 5.0 mmol/L. Incorporated in these closed-loop systems is a matrix that contains insulin with a glucose-sensing element. When there is an increase in BGLs, the matrix conformation alters, resulting in GRIR, and returns to its original structural conformation when BGLs return to normal and insulin is no longer released [102]. With the glucose-sensing element, insulin release mechanisms exist and include matrix swelling, disassembly or degradation, and glucose-binding competition. These controlled release mechanisms are illustrated in Figure 7. These systems allow for personalized healthcare and vastly improved control of BGLs [12]. There are three groups that have been developed for chemically controlled closed-loop systems, such as glucose oxidase, PBA, and Concanavalin A [49]. These systems will be discussed in detail below.

### 4.1. Glucose Oxidase (GOx)

GOx, a glucose-specific enzyme, is commonly used for blood glucose quantification in glucometers. In stimuli-responsive systems, as BGLs increase, GOx causes glucose to be broken down into gluconic acid. This reaction takes place via oxidation, with hydrogen peroxide being an end product, as shown in Equations (1) and (2) below [104]. The formation of gluconic acid causes a drop in physiological pH levels, stimulating the swelling or disassembly of the pH system, thus resulting in the controlled release of insulin [102]:

(1) $$D-glucose+O_{2}\to D-gluconolactone+\;H_{2}O_{2}$$

(2) $$D-gluconolactone+H_{2}O\to D-gluconic\;acid$$

**Figure 8:**
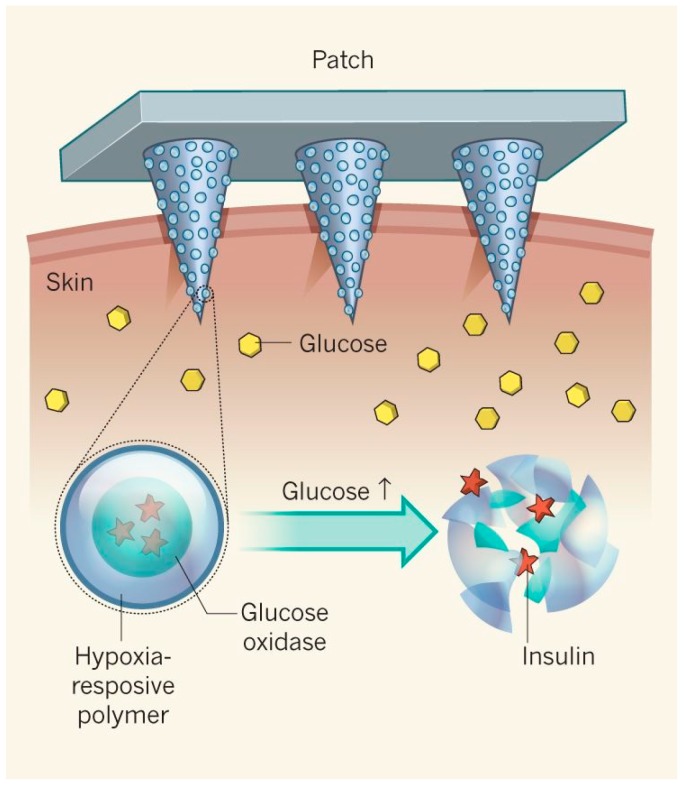
Illustration of an insulin patch and stimuli-responsive mechanism for transdermal insulin delivery (reproduced with permission from Reference [106]).

A “smart insulin patch” was fabricated and analyzed by Yu et al. The researchers designed an HA crosslinked matrix with hypoxia-responsive vesicles for the synthesis of a glucose-responsive microneedle patch. NPs were embedded within the needles and consisted of insulin, the enzyme, glucose oxidase, and a polymer that is responsive to hypoxic conditions (Figure 8). The results reported that glucose-responsive vesicles were able to release insulin rapidly when BGLs increased and lower BGLs to 200 mg/dL within 0.5 h in vivo. BGLs were able to be maintained without hypoglycemic effects when another patch was administered. [105,106]

Gu et al. synthesized alginate-coated, dextran, and CS pH-responsive NPs via electrostatic interactions. The NPs degraded at an acidic pH via glucose reaction with GOx. A bioresponsive profile was shown in response to glucose concentrations, while animal studies demonstrated the ability of the NPs to stabilize BGLs over 10 days [107]. Glucose-responsive insulin release neoglycoenzyme-capped MSNs were developed by Oroval et al. Propylbenzimidazole moieties were integrated into the MSN, while β-cyclodextran/GOx was used to cap the MSN. Insulin was labeled with FITC (Ins-FITC). When the MSN was uncapped, via gluconic acid protonation by the benzimidazole group, the insulin FITC dye was released. Using a gated mesoporous silica nanodevice, glucose-responsive Ins-FITC release was evaluated. The results showed that β-cyclodextran/GOx was uncapped from the MSN, thereby releasing Ins-FITC, with insulin activity being maintained. The system was highly selective, with insulin release being stimulated by glucose only (no other saccharides were evaluated) [108]. It must be noted that GOx is highly responsive to changes in its environment and can thus be denatured when entrapped in or covalently linked to a polymeric matrix [108,109].

### 4.2. Phenylboronic Acid (PBA)

As a glucose-binding molecule, PBA is able to form covalent complexes that are reversible with glucose and other polyol molecules. PBA moieties, when in equilibrium, demonstrate two structural forms: a charged hydrophilic form and an uncharged hydrophobic form. When BGLs rise, the hydrophilic form can develop a stable complex with glucose. This takes place via reversible covalent bonding. Once the stable complex is formed, equilibrium is shifted toward increasing hydrophilic PBA forms. PBA closed-loop GRIR systems are able to be incorporated into a multitude of platforms, including nanotechnology. Due to its small size, the PBA molecule is able to be used in multiple chemical modifications in the design of the closed-loop system. However, the PBA mechanism cannot function at a physiological pH and instead works best in a basic environment due to its high pKa value. Additionally, the low specificity of PBA toward glucose influences its accuracy and insulin release response time negatively. The toxicity of this system is not well established [102]. Zhang and coworkers prepared layer-by-layer films consisting of poly (acrylamide) copolymer with PBA side chains (PBA-PAAm) and PVA, in which the PBA moieties of PBA-PAAm and the 1,3-diol units of PVA formed boronate ester bonds [110]. The bilayered film demonstrated good glucose sensitivity in the presence of glucose and degraded by 25% after 200 h in vitro. In another study, Wang et al. developed shell crosslinked NPs via the complexation of poly(3 methacrylamido PBA) (PMAPBA) and thiolated CS (CS-SH) through boronic acid-related reactions. The release of insulin was affected by glucose levels in the medium [111].

A shortfall of this system is that most PBA-based GRIR systems cannot function at a physiological pH, but rather at an alkaline pH, due to the PBA moieties’ high pKa value [112]. PBA also has a poor specificity to glucose, which has an effect on the accuracy and response time of the fabricated system [113].

### 4.3. Concanavalin A (ConA)

**Figure 9:**
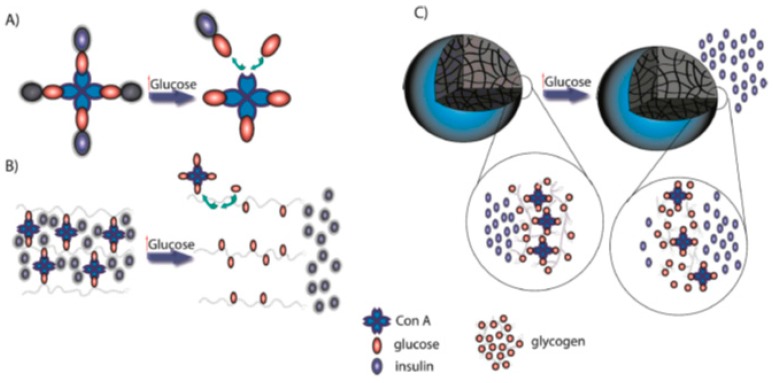
Schematic representing (**A**) ConA, with an affinity to glucose, with four binding sites for glucose or glucose-linked molecules. (**B**) When an increase in blood glucose levels (BGLs) occurs, ConA binds to free glucose and releases its bonds to the matrix polymer, resulting in a separation of the polymeric chain. The hydrogel goes from gel to sol, with insulin being released. (**C**) With high BGLs, attachments between ConA and glycogen are destroyed, and the outer part of the carrier swells, with insulin being released (adapted with permission from Reference [4]).

Lectins are a family of glucose-binding proteins. They are derived from the jack bean plant and are naturally able to bind to carbohydrates [114]. ConA is the most commonly used lectin. ConA is a tetramer with four sugar binding sites, demonstrating a strong affinity to glucose. It can be used as a biosensor in the development of stimuli-responsive systems. [115]. When BGLs increase, glucose molecules compete for existing binding between polysaccharides and ConA [116]. The use of polymers in ConA stimuli-responsive systems has been explored. The incorporation of ConA in nanosphere fabrication, as well as in attachments to polymers, has been investigated for the sustained release of insulin in response to glucose. Reversible binding to ConA is possible when the glycosylation of insulin takes place. When this happens, competitive binding to ConA occurs between glucose and the glycosylated portion i.e., the ConA-glycosylated insulin bond breaks apart, as seen in Figure 9 [117]. When BGLs rise, the equilibrium shifts to the free glycosylated insulin. However, the use of ConA has limitations. Its toxicity, stability, and aqueous solubility are challenges that may be improved with the use of hydrophilic polymers [109]. Studies carried out for the development of stimuli-responsive NPs at Massachusetts Institute of Technology MIT, Cambridge, showed that a ConA/FITC dextran system was encouraging. The crosslinked NPs were fabricated via reverse microemulsion, demonstrating insulin released in response to glucose changing with the ConA/dextran ratio [118]. Chang et al. prepared stimuli-responsive biopolymer nanocarriers using ConA and amylopectin. Fluorimetry data showed that different concentrations of amylopectin altered the ConA conformation. Insulin was successfully encapsulated in the nanocarriers, with an encapsulation efficiency and loading capacity of 69.73% and 17%, respectively. The NPs had an insulin release rate that was 2.33 times more when NPs were in a glucose environment as opposed to a nonglucose environment [119]. Researchers in India fabricated ConA surface-modified PLGA NPs for the oral delivery of insulin. The PLGA insulin-loaded NPs were prepared via a double emulsion solvent evaporation method conjugated via EDC/NHS coupling. In vitro studies demonstrated a sustained release for one day with a 58.1% insulin release. Animal studies were also carried out on STZ-induced diabetic Wistar rats. The results demonstrated the effective lowering of BGLs to 62.17 mg/dL within 4 h of administration [120].

A novel ConA/poly (NIPAM) polymer nanogel was developed by Ye et al. via free radical precipitation copolymerization. In order to study insulin release, normoglycemic and hyperglycemic environments were stimulated with the nanogel in dialysis tubing. A basal and bolus insulin release was achieved along with a controlled insulin release over 48 h. The affinity of ConA to glucose has allowed for the development of multiple stimuli-responsive platforms for insulin delivery [121]. Additionally, studies carried out by Yin et al. demonstrated that ConA micro/nanospheres could be embedded in a polymer matrix, as illustrated in Figure 2, for ideal insulin therapy. However, more work needs to be carried out, as this was undertaken in vitro only [49].

## 5. Future Perspective for Insulin Nanotherapeutics

Novel insulin formulations with alternative routes of administration have also been taken to clinical trials. Insulin Pharmfilm went into stage 2a clinical trials in 2015. Pharmfilm was developed by MonoSol Rx and Midatech as a transbuccal mucoadhesive film. Unfortunately, the drug release profile was poor [122]. Other platforms that show promise in insulin delivery are porous microparticles. They are fabricated via the supercritical fluid process, in addition to spray drying, milling, and emulsion solvent evaporation [123]. The porous microspheres can be used for the pulmonary administration of insulin, as they are able to avoid an immune response due to their size and penetrate deep in the alveoli. Although the platform is at a microscale, polymers such as CS are able to be employed at a nanoscale for drug administration [124]. Current research aims to achieve oral insulin delivery. However, building on nanotechnology and polymer chemistry, the development of glucose-responsive nano-insulin delivery systems holds much promise for the future. It is this system that satisfies the goals of insulin delivery most aptly. The closed-loop system will release insulin in an appropriate dose response manner over a prolonged period of time in a self-administrative manner that is minimally invasive, thereby improving patient compliance [114]. Although not in clinical stages yet, advancements in bionanotechnology will overcome the challenges to smart insulin delivery systems [4]. Nevertheless, it is important that stimuli-responsive systems are appropriately assessed for biocompatibility to achieve a prolonged therapy for the treatment of chronic diabetes with minimal side effects [114]. An ideal insulin therapy must be minimally invasive, self-administrable, biodegradable, and biocompatible, with prolonged release responding to changes in BGLs.

## 6. Conclusions

Insulin has played a pivotal role in the treatment of DM to date. Subsequently, insulin therapy has undergone substantial progress, improved by the incorporation of nanotechnology into multiple platforms and the molecular manipulation of insulin for sustained therapeutic effects. The polymeric incorporation of insulin, using natural and synthetic biopolymers, has expanded the possibility of many routes for efficient insulin delivery. To date, research avenues of oral, buccal, nasal, pulmonary, and transdermal means of insulin delivery have been explored with exponential progress. The oral route of administration for insulin has seen the most significant advances in comparison to other routes, employing stimuli-responsive polymeric platforms for controlled insulin delivery. Since the oral route is minimally invasive and self-administrable, it constitutes the most convenient and socially accepted platform for therapeutic insulin delivery. The advances made in NP formulations in all platforms of nano-insulin delivery have contributed significantly to cutting edge research, delivering high bioavailable doses of insulin with the aid of nanopolymeric systems.

## Figures and Tables

**Table 1:** Summary of natural and synthetic polymers, their advantages, polymeric complexes, delivery systems, and alternative routes of administration employed in nano-insulin delivery. NPs: nanoparticles.

Polymer	Advantages	Polymeric Complex and Delivery System	Route of Administration
**Natural**			
CS	- Abundant in nature - Mucoadhesive - Biodegradable - Nontoxic - Inexpensive	- Trimethyl chitosan (TMC) NPs [5] - Chitosan (CS)/reduced gold (Au) nanoparticles (NPs) [13] - CS-g-polyethylene glycol monomethyl ether (mPEG) NP nanocomplex [14] - CS polyelectrolyte (PEC) NPs [15] - CS NPs [16]	- Oral [5,13,14,15] - Nasal [13,16]
Alginate	- Derived from nature - Polyanionic - Biodegradable - Nontoxic - Mucoadhesive - Low immunogenicity - Able to form a gel at acidic pH or if divalent cations are present	- Alginate/CS/β-cyclodextran NPs [17] - Alginate/CS PEC NPs [18] - Alginate/CS-coated NPs [19] - Calcium phosphate-coated alginate/CS/Vitamin B12 (VB12) NPs [20]	- Oral [17,18,19,20]
HA	- Biocompatible - Biodegradable - Low immunogenicity	- Hyaluronic Acid (HA)-coated calcium carbonate NPs [21] - HA NPs [22]	- Oral [21,22]
Dextran	- Biodegradable - Biocompatible - Hydrophilic - OH functional groups allow for a variety of manipulations	- Dextran/alginate sulfate NPs with CS/albumin coating [23] - Dextran/ Poly-Lactic-co-Glycolic Acid (PLGA) NPs [24] - VB12/intrinsic factor conjugates on dextran NPs [25]	- Oral [23,24,25]
Gelatin	- Biodegradable - Nontoxic - Hydrophilic - Polyampholyte - Crosslinking potential	- Gelatin/glutaraldehyde NPs [26] - Gelatin/poloxamer NPs [27]	- Oral [26] - Pulmonary [27]
**Synthetic**			
PLGA	- Biodegradable - Controlled release kinetics	- Zinc insulin in PLGA NPs [28] - PLGA NPs [29] - PLGA-conjugated CS NPs [30] - PLGA NPs within polyvinyl alcohol PVA hydrogel [31]	- Oral [28,29,30] - Intraperitoneal injection [31]
PCL	- Biodegradable - Degrade over a period of time - Mucoadhesive	- Poly-ε-Caprolactone (PCL) NPs [32] - PCL-blended cationic acrylic NPs [33] - mPEG/PCL/ *N*, *N*-diethylamino-2-ethylmathaerylate (PDEAEMA) NPs [34]	- Oral [32,33]
PVA	- Biocompatible - Biodegradable - Low toxicity - Thermal stability - High level of mechanical strength - Blended (natural polymers)	- PVA/CS nano-insulin-loaded hydrogels [35] - PVA NPs [36]	- Transdermal [35] - Oral [36]
Polyamino Acids	- Chirality - Reversible crosslinking - Hydrophilic - Hydrophobic - Charge density	- CS/poly-g-glutamic acid NPs [37] - Gelatin-coated CS/poly (γ-glutamic acid) NPs [38] - L valine/PBA NPs [39]	- Oral [38,39]
Pluronic	- Biodegradable - Amphiphilic - Thermosensitive properties - Soluble in aqueous, polar, and nonpolar solvents	- Poly lactic acid (PLA)/pluronic/PLA NPs [40] - Folic acid/pluronic/PLGA NPs [41]	- Oral [40,41]

**Table 2:** Summary of advantages and challenges associated with polymeric nanoplatforms for various insulin delivery approaches.

Delivery Approach	Advantages	Challenges	Polymeric Systems	References
Oral	- Improved patient compliance - Closely mimics physiological insulin route - Good glucose homeostasis	- Acidic pH of GI - Degradation by proteolytic enzymes - Low permeation through intestine	Nanoparticles (NPs) Solid Lipid Nanoparticles (SLNs) Cell Penetrating Peptides (CPP) Nanogel	[6,44,63,64,65,66,67,68,69,70,71,72]
Buccal	- Large surface area - Easy and painless administration - Permeation enhancers/enzymes can be incorporated	- Mucosal membranes may prevent drug from reaching systemic circulation - Taste of drug system may result in poor patient compliance - Accidental swallowing may occur	Transfersomes	[53,73]
Nasal	- Easy introduction to systemic circulation - Avoids gastric degradation	- Nasal mucosa barrier for insulin - Mucociliary clearance	NPs Dendrimers	[53,64,74,75]
Pulmonary	- Large surface area of alveolar epithelium resulting in quick systemic circulation	- Pulmonary administration may activate an immune response - Mucosal barriers	Dendrimers	[53,54,76]
Transdermal	- Sustained release - Minimally invasive - Avoidance of gastrointestinal tract (GIT)	- Skin barrier - Epidermis limits drug diffusion to systemic circulation	NPs Transfersomes	[53,55,77,78,79]
